# 20,23,26,29,32,35,38,41-Octa­oxa-5,9,13-triaza­penta­cyclo­[15.14.10.1^3,30^.1^7,11^.1^15,19^]tetra­tetra­conta-1,3(42),7,9,11(44),15(43),16,18,30-nona­ene-6,12-dione acetone mono­solvate

**DOI:** 10.1107/S1600536811051877

**Published:** 2011-12-14

**Authors:** Shaowu Pan, Dengke Yang, Yu Yang, Lasheng Jiang

**Affiliations:** a School of Chemistry and Environment, South China Normal University, Guangzhou 510006, People’s Republic of China

## Abstract

In the crystal structure of the title compound, C_33_H_39_N_3_O_10_·C_3_H_6_O,  the acetone mol­ecule is encapsulated into the cavity of the cryptand and fixed by two N—H⋯O and one C—H⋯O hydrogen bond. C—H⋯O and C—H⋯N inter­actions link neighbouring cryptands. The dihedral angles between the pyridine ring and the benzene rings are 86.47 (17) and 85.53 (13)°.

## Related literature

Cryptands have been utilized as hosts to form supra­molecular assemblies, see: Balzani *et al.* (2000 ▶). Crown ether-based cryptands can form more stable supra­molecular complexes with paraquat, paraquat derivatives, diquat and secondary ammonium salts than the corresponding simple crown ethers by virtue of multiple non-covalent inter­actions, see: Huang *et al.* (2005 ▶). The title compound was obtained by the reaction of bis­(5-amino­methyl-1,3-phenyl­ene)-26-crown-8 (Wester & Voegtle, 1980 ▶) with pyridine-3,5-dicarbonyl dichloride (Chen *et al.*, 2010 ▶).
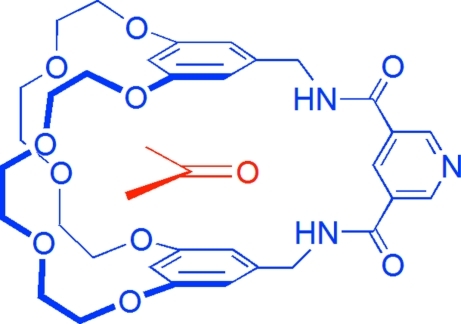


## Experimental

### 

#### Crystal data

C_33_H_39_N_3_O_10_·C_3_H_6_O
*M* *_r_* = 695.75
Tetragonal, 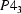

*a* = 14.232 (3) Å
*c* = 17.615 (4) Å
*V* = 3567.8 (12) Å^3^
*Z* = 4
Mo *K*α radiation
μ = 0.10 mm^−1^
*T* = 298 K
0.32 × 0.28 × 0.25 mm

#### Data collection

Bruker APEXII CCD area-detector diffractometer
Absorption correction: multi-scan (*SADABS*; Sheldrick, 1996 ▶) *T*_min_ = 0.970, *T*_max_ = 0.976
19761 measured reflections
3626 independent reflections
2127 reflections with *I* > 2σ(*I*)
*R*_int_ = 0.059

#### Refinement

*R*[*F* ^2^ > 2σ(*F* ^2^)] = 0.056
*wR*(*F* ^2^) = 0.172
*S* = 1.00
3626 reflections
453 parameters
1 restraint
H-atom parameters constrained
Δρ_max_ = 0.33 e Å^−3^
Δρ_min_ = −0.19 e Å^−3^

### 

Data collection: *APEX2* (Bruker, 2008 ▶); cell refinement: *SAINT* (Bruker, 2008 ▶); data reduction: *SAINT*; program(s) used to solve structure: *SHELXS97* (Sheldrick, 2008 ▶); program(s) used to refine structure: *SHELXL97* (Sheldrick, 2008 ▶); molecular graphics: *SHELXTL* (Sheldrick, 2008 ▶); software used to prepare material for publication: *SHELXTL*.

## Supplementary Material

Crystal structure: contains datablock(s) global, I. DOI: 10.1107/S1600536811051877/aa2033sup1.cif

Structure factors: contains datablock(s) I. DOI: 10.1107/S1600536811051877/aa2033Isup2.hkl

Supplementary material file. DOI: 10.1107/S1600536811051877/aa2033Isup3.cml

Additional supplementary materials:  crystallographic information; 3D view; checkCIF report

## Figures and Tables

**Table 1:** Hydrogen-bond geometry (Å, °)

*D*—H⋯*A*	*D*—H	H⋯*A*	*D*⋯*A*	*D*—H⋯*A*
N1—H1⋯O1	0.86	2.36	3.209 (7)	170
N2—H2⋯O1	0.86	2.53	3.382 (7)	172
C9—H9⋯O1	0.93	2.43	3.271 (7)	151
C3—H3⋯O11^i^	0.93	2.46	3.356 (6)	161
C33—H33*B*⋯N3^ii^	0.97	2.60	3.401 (8)	140

Symmetry codes: (i) 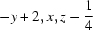; (ii) 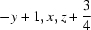.

**Fig. 1.**
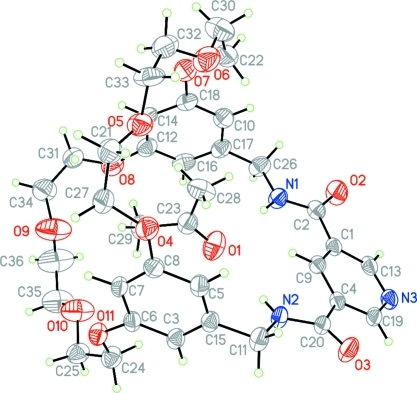
View of the title compound showing the atom-labelling scheme. Ellipsoids are drawn at the 30% probability level.

**Fig. 2.**
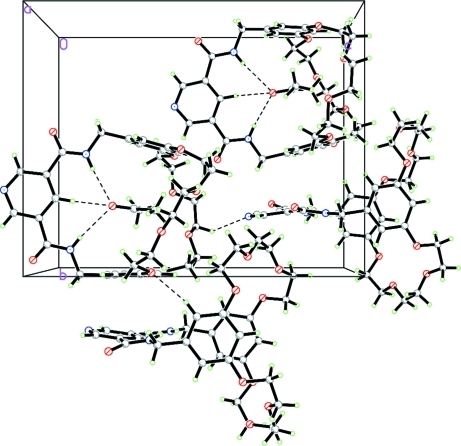
Perspective view of the crystal packing.

## supplementary crystallographic information

### Comment

The chemistry of inclusion complexes has been widely investigated by scientists all over the world with various applications. Cryptands have been universally utilized as hosts to form supramolecular assemblies, which have great potential applications in molecular devices and material science (Balzani *et al.*, 2000). Recently, crown ether-based cryptands have attracted much attention due to their ability to form more stable supramolecular complexes with paraquat, paraquat derivatives, diquat and secondary ammonium salts than corresponding simple crown ethers by virtue of multiple noncovalent interactions (Huang *et al.*, 2005). We are interested in developing novel crown ether-based cryptands and their applications in supramolecular self-assembly. Herein, we report a novel crown ether-based cryptand and its crystal structure, which was obtained by the reaction of bis(5-aminomethyl-1,3-phenylene)-26-crown-8 (Wester & Voegtle, 1980) with pyridine-3,5-dicarbonyl dichloride (Chen *et al.*, 2010).

The crystal structure of the title compound is illustrated in Fig.1. The dihedral angle between the benzene ring of C17/C10/C18/C14/C12/C16 and the benzene ring of C15/C5/C8/C7/C6/C3 is 15.6 (3)°, which means the two benzene rings are approximately parallel. So it is advantageous to involve small organic compounds. The acetone molecule forms three hydrogen bonds to the cryptand (see Table 1).

### Experimental

To a stirred solution of triethylamine in dichloromethane (120 ml), was added bis(5-aminomethyl-1,3-phenylene)-26-crown-8 (253 mg, 0.5 mmol) in dichloromethane (20 ml) and pyridine-3,5-dicarbonyl dichloride (102 mg, 0.5 mmol) in dichloromethane (20 ml) simultaneously with two pressure-equalizing dropping funnels at 5 ml/h. After addition, the reaction mixture was strirred for 18 h at room temperature. Water (30 ml) was added, and then the resulting mixture was neutralized and extracted with dichloromethane. The organic layer was dried over anhydrous MgSO_4_ and concentrated. The crude product was separated by column chromatography to give the desired cryptand (64 mg, 20%) as a white solid. The crystal was obtained by slow evaporation of mixed solvent of dichloromethane and acetone at room temperature.

### Refinement

All H atoms were fixed geometrically and were treated as riding on their parent C and N atoms, with C—H distances in the range of 0.93–0.97 Å, N—H = 0.86 Å, and with *U*_iso_(H) = 1.5 *U*_eq_(C) for methyl H atoms and *U*_iso_(H) = 1.2 *U*_eq_(C,N) for other H atoms.

### Crystal data

**Table:**

C_33_H_39_N_3_O_10_·C_3_H_6_O	*D*_x_ = 1.295 Mg m^−^^3^
*M**_r_* = 695.75	Mo *K*α radiation, λ = 0.71073 Å
Tetragonal, *P*4_3_	Cell parameters from 19761 reflections
Hall symbol: P 4cw	θ = 1.4–26.0°
*a* = 14.232 (3) Å	µ = 0.10 mm^−^^1^
*c* = 17.615 (4) Å	*T* = 298 K
*V* = 3567.8 (12) Å^3^	Block, colourless
*Z* = 4	0.32 × 0.28 × 0.25 mm
*F*(000) = 1480	

### Data collection

**Table:**

Bruker APEXII CCD area-detector diffractometer	3626 independent reflections
Radiation source: fine-focus sealed tube	2127 reflections with *I* > 2σ(*I*)
graphite	*R*_int_ = 0.059
phi and ω scans	θ_max_ = 26.0°, θ_min_ = 1.4°
Absorption correction: multi-scan (*SADABS*; Sheldrick, 1996)	*h* = −17→16
*T*_min_ = 0.970, *T*_max_ = 0.976	*k* = −17→17
19761 measured reflections	*l* = −21→13

### Refinement

**Table:**

Refinement on *F*^2^	Primary atom site location: structure-invariant direct methods
Least-squares matrix: full	Secondary atom site location: difference Fourier map
*R*[*F*^2^ > 2σ(*F*^2^)] = 0.056	Hydrogen site location: inferred from neighbouring sites
*wR*(*F*^2^) = 0.172	H-atom parameters constrained
*S* = 1.00	*w* = 1/[σ^2^(*F*_o_^2^) + (0.1014*P*)^2^] where *P* = (*F*_o_^2^ + 2*F*_c_^2^)/3
3626 reflections	(Δ/σ)_max_ = 0.002
453 parameters	Δρ_max_ = 0.33 e Å^−^^3^
1 restraint	Δρ_min_ = −0.19 e Å^−^^3^

### Special details

**Table:**

Geometry. All s.u.'s (except the s.u. in the dihedral angle between two l.s. planes) are estimated using the full covariance matrix. The cell s.u.'s are taken into account individually in the estimation of s.u.'s in distances, angles and torsion angles; correlations between s.u.'s in cell parameters are only used when they are defined by crystal symmetry. An approximate (isotropic) treatment of cell s.u.'s is used for estimating s.u.'s involving l.s. planes.
Refinement. Refinement of *F*^2^ against ALL reflections. The weighted *R*-factor *wR* and goodness of fit *S* are based on *F*^2^, conventional *R*-factors *R* are based on *F*, with *F* set to zero for negative *F*^2^. The threshold expression of *F*^2^ > σ(*F*^2^) is used only for calculating *R*-factors(gt) *etc*. and is not relevant to the choice of reflections for refinement. *R*-factors based on *F*^2^ are statistically about twice as large as those based on *F*, and *R*- factors based on ALL data will be even larger. Since this is a light atom structure (it does not contain any atoms heavier than Si) and since the data collection was carried out using Mo radiation, it is impossible to unambiguously determine the absolute configuration of this molecule.

### Fractional atomic coordinates and isotropic or equivalent isotropic displacement parameters (Å^2^)

**Table:**

	*x*	*y*	*z*	*U*_iso_*/*U*_eq_	
O1	0.8054 (4)	0.7413 (4)	0.2477 (3)	0.1119 (17)	
O2	0.7434 (4)	0.4588 (3)	0.0601 (3)	0.0993 (15)	
O3	0.7236 (3)	0.9474 (3)	−0.0035 (2)	0.0830 (12)	
O11	0.9501 (2)	0.9872 (3)	0.37266 (19)	0.0658 (10)	
O4	0.6174 (3)	0.9965 (3)	0.3937 (2)	0.0740 (10)	
O7	0.5654 (3)	0.5410 (3)	0.4265 (3)	0.0881 (13)	
O8	0.8974 (3)	0.5340 (3)	0.4588 (3)	0.0912 (13)	
O5	0.5101 (3)	0.8513 (3)	0.4713 (2)	0.0828 (12)	
O6	0.4182 (4)	0.6829 (3)	0.4163 (3)	0.1031 (15)	
O9	1.0150 (4)	0.6896 (4)	0.5070 (5)	0.141 (3)	
O10	1.0690 (3)	0.8355 (4)	0.4078 (4)	0.127 (2)	
N2	0.7616 (3)	0.9071 (3)	0.1148 (2)	0.0578 (10)	
H2	0.7766	0.8625	0.1455	0.069*	
N1	0.7660 (3)	0.5452 (3)	0.1635 (3)	0.0701 (12)	
H1	0.7701	0.6003	0.1834	0.084*	
N3	0.7796 (3)	0.6805 (4)	−0.0855 (2)	0.0668 (12)	
C1	0.7603 (3)	0.6219 (4)	0.0422 (3)	0.0546 (12)	
C2	0.7553 (4)	0.5357 (4)	0.0897 (3)	0.0584 (13)	
C3	0.8547 (4)	0.9978 (3)	0.2587 (3)	0.0568 (13)	
H3	0.9070	0.9959	0.2271	0.068*	
C4	0.7574 (3)	0.7870 (3)	0.0188 (3)	0.0495 (11)	
C5	0.6878 (4)	1.0033 (3)	0.2752 (3)	0.0579 (13)	
H5	0.6277	1.0054	0.2544	0.070*	
C6	0.8652 (4)	0.9957 (3)	0.3360 (3)	0.0545 (12)	
C7	0.7876 (3)	0.9989 (3)	0.3843 (3)	0.0545 (12)	
H7	0.7957	1.0000	0.4367	0.065*	
C8	0.6990 (4)	1.0006 (3)	0.3530 (3)	0.0578 (13)	
C9	0.7524 (3)	0.7122 (3)	0.0686 (3)	0.0511 (12)	
H9	0.7438	0.7230	0.1202	0.061*	
C10	0.6657 (4)	0.4984 (4)	0.3207 (4)	0.0701 (15)	
H10	0.6147	0.4905	0.2884	0.084*	
C11	0.7524 (4)	1.0015 (4)	0.1435 (3)	0.0670 (14)	
H11A	0.6909	1.0260	0.1305	0.080*	
H11B	0.7995	1.0414	0.1201	0.080*	
C12	0.8172 (4)	0.5212 (4)	0.4178 (4)	0.0719 (15)	
C13	0.7736 (3)	0.6112 (4)	−0.0360 (3)	0.0608 (13)	
H13	0.7787	0.5503	−0.0546	0.073*	
C14	0.7273 (4)	0.5348 (4)	0.4452 (3)	0.0708 (15)	
H14	0.7173	0.5515	0.4956	0.085*	
C15	0.7646 (4)	1.0030 (3)	0.2283 (3)	0.0576 (12)	
C16	0.8306 (5)	0.4957 (4)	0.3418 (4)	0.0747 (16)	
H16	0.8910	0.4857	0.3234	0.090*	
C17	0.7550 (5)	0.4857 (4)	0.2943 (3)	0.0679 (15)	
C18	0.6518 (4)	0.5231 (4)	0.3952 (3)	0.0676 (14)	
C19	0.7702 (3)	0.7669 (4)	−0.0574 (3)	0.0573 (13)	
H19	0.7724	0.8171	−0.0911	0.069*	
C20	0.7474 (3)	0.8878 (4)	0.0420 (3)	0.0536 (12)	
C21	0.5283 (4)	0.9426 (4)	0.4984 (4)	0.0808 (17)	
H21A	0.5255	0.9432	0.5535	0.097*	
H21B	0.4813	0.9858	0.4792	0.097*	
C22	0.4860 (5)	0.5395 (5)	0.3770 (4)	0.0870 (18)	
H22A	0.4709	0.4751	0.3638	0.104*	
H22B	0.5009	0.5730	0.3306	0.104*	
C23	0.7716 (4)	0.7518 (4)	0.3107 (3)	0.0712 (15)	
C24	1.0285 (4)	0.9579 (4)	0.3280 (3)	0.0741 (16)	
H24A	1.0528	1.0108	0.2994	0.089*	
H24B	1.0088	0.9099	0.2923	0.089*	
C25	1.1021 (4)	0.9204 (5)	0.3775 (4)	0.0819 (17)	
H25A	1.1593	0.9098	0.3488	0.098*	
H25B	1.1157	0.9645	0.4181	0.098*	
C26	0.7707 (5)	0.4622 (4)	0.2112 (3)	0.0782 (17)	
H26A	0.8319	0.4329	0.2054	0.094*	
H26B	0.7235	0.4174	0.1948	0.094*	
C27	0.6231 (4)	0.9722 (5)	0.4727 (3)	0.0779 (16)	
H27A	0.6443	1.0258	0.5020	0.093*	
H27B	0.6677	0.9213	0.4799	0.093*	
C28	0.6695 (5)	0.7563 (5)	0.3211 (5)	0.100 (2)	
H28A	0.6486	0.8199	0.3145	0.150*	
H28B	0.6538	0.7352	0.3713	0.150*	
H28C	0.6394	0.7167	0.2843	0.150*	
C29	0.8342 (5)	0.7579 (4)	0.3779 (4)	0.0857 (19)	
H29A	0.8323	0.6996	0.4052	0.129*	
H29B	0.8134	0.8080	0.4103	0.129*	
H29C	0.8973	0.7702	0.3615	0.129*	
C30	0.4058 (5)	0.5828 (5)	0.4130 (5)	0.105 (2)	
H30A	0.3493	0.5681	0.3846	0.126*	
H30B	0.3986	0.5580	0.4640	0.126*	
C31	0.8920 (5)	0.5784 (5)	0.5309 (4)	0.0841 (18)	
H31A	0.8689	0.5344	0.5685	0.101*	
H31B	0.8490	0.6312	0.5286	0.101*	
C32	0.4120 (6)	0.7166 (5)	0.4873 (4)	0.110 (3)	
H32A	0.4615	0.6887	0.5177	0.132*	
H32B	0.3523	0.6974	0.5088	0.132*	
C33	0.4196 (5)	0.8199 (5)	0.4922 (4)	0.095 (2)	
H33A	0.3733	0.8484	0.4591	0.114*	
H33B	0.4063	0.8397	0.5438	0.114*	
C34	0.9884 (5)	0.6118 (5)	0.5524 (5)	0.103 (2)	
H34A	0.9889	0.6299	0.6055	0.123*	
H34B	1.0332	0.5612	0.5456	0.123*	
C35	1.1288 (5)	0.7776 (6)	0.4430 (6)	0.118 (3)	
H35A	1.1599	0.8135	0.4825	0.142*	
H35B	1.1767	0.7600	0.4064	0.142*	
C36	1.0958 (6)	0.6952 (6)	0.4760 (7)	0.142 (4)	
H36A	1.0979	0.6471	0.4370	0.170*	
H36B	1.1413	0.6772	0.5143	0.170*	

### Atomic displacement parameters (Å^2^)

**Table:**

	*U*^11^	*U*^22^	*U*^33^	*U*^12^	*U*^13^	*U*^23^
O1	0.133 (4)	0.135 (4)	0.068 (3)	0.017 (3)	0.006 (3)	−0.015 (3)
O2	0.150 (4)	0.059 (3)	0.089 (3)	−0.006 (2)	−0.010 (3)	−0.023 (2)
O3	0.129 (4)	0.069 (3)	0.051 (2)	0.004 (2)	−0.012 (2)	0.010 (2)
O11	0.065 (2)	0.080 (2)	0.052 (2)	0.0050 (18)	−0.0022 (18)	−0.0076 (18)
O4	0.065 (2)	0.094 (3)	0.063 (3)	−0.0021 (19)	0.0052 (19)	−0.002 (2)
O7	0.084 (3)	0.106 (3)	0.075 (3)	−0.008 (2)	0.011 (2)	−0.010 (2)
O8	0.082 (3)	0.106 (3)	0.086 (3)	0.010 (2)	0.001 (2)	0.014 (3)
O5	0.089 (3)	0.083 (3)	0.077 (3)	−0.011 (2)	0.027 (2)	−0.016 (2)
O6	0.134 (4)	0.090 (3)	0.085 (3)	−0.011 (3)	0.025 (3)	−0.012 (3)
O9	0.090 (4)	0.117 (4)	0.216 (7)	0.007 (3)	0.001 (4)	0.079 (5)
O10	0.081 (3)	0.121 (4)	0.179 (6)	0.004 (3)	0.000 (3)	0.063 (4)
N2	0.083 (3)	0.048 (2)	0.042 (2)	0.0077 (19)	−0.012 (2)	−0.0021 (18)
N1	0.102 (4)	0.052 (3)	0.056 (3)	−0.003 (2)	0.003 (2)	−0.007 (2)
N3	0.065 (3)	0.091 (3)	0.045 (2)	−0.013 (2)	0.004 (2)	−0.018 (3)
C1	0.049 (3)	0.068 (3)	0.047 (3)	−0.008 (2)	0.003 (2)	−0.016 (2)
C2	0.063 (3)	0.049 (3)	0.063 (4)	0.003 (2)	0.001 (3)	−0.017 (3)
C3	0.076 (3)	0.050 (3)	0.044 (3)	0.003 (2)	0.000 (2)	−0.006 (2)
C4	0.040 (2)	0.064 (3)	0.044 (3)	−0.001 (2)	−0.003 (2)	−0.009 (2)
C5	0.072 (3)	0.047 (3)	0.055 (3)	0.002 (2)	−0.009 (3)	−0.001 (2)
C6	0.068 (3)	0.047 (3)	0.048 (3)	0.002 (2)	−0.008 (2)	−0.002 (2)
C7	0.067 (3)	0.056 (3)	0.041 (3)	−0.003 (2)	−0.005 (2)	−0.004 (2)
C8	0.065 (3)	0.050 (3)	0.059 (3)	−0.001 (2)	0.000 (3)	−0.002 (2)
C9	0.049 (3)	0.061 (3)	0.043 (3)	−0.001 (2)	0.007 (2)	−0.010 (2)
C10	0.085 (4)	0.054 (3)	0.072 (4)	−0.003 (3)	0.003 (3)	−0.004 (3)
C11	0.099 (4)	0.049 (3)	0.053 (3)	0.011 (3)	−0.010 (3)	−0.004 (2)
C12	0.077 (4)	0.071 (4)	0.068 (4)	0.000 (3)	−0.001 (3)	0.016 (3)
C13	0.054 (3)	0.075 (4)	0.053 (3)	−0.010 (2)	−0.001 (2)	−0.020 (3)
C14	0.092 (4)	0.065 (3)	0.056 (3)	−0.002 (3)	0.012 (3)	0.010 (3)
C15	0.080 (4)	0.046 (3)	0.047 (3)	0.000 (2)	−0.007 (3)	−0.003 (2)
C16	0.080 (4)	0.060 (3)	0.084 (5)	0.004 (3)	0.017 (4)	0.015 (3)
C17	0.093 (4)	0.049 (3)	0.062 (4)	0.003 (3)	0.011 (3)	0.006 (2)
C18	0.072 (4)	0.065 (3)	0.066 (4)	−0.004 (3)	0.017 (3)	0.007 (3)
C19	0.052 (3)	0.081 (4)	0.039 (3)	−0.006 (2)	0.002 (2)	−0.002 (3)
C20	0.056 (3)	0.065 (3)	0.040 (3)	−0.005 (2)	−0.001 (2)	0.000 (2)
C21	0.083 (4)	0.088 (4)	0.071 (4)	−0.004 (3)	0.016 (3)	−0.018 (3)
C22	0.081 (4)	0.092 (4)	0.089 (5)	−0.013 (3)	0.008 (4)	−0.015 (4)
C23	0.091 (4)	0.056 (3)	0.066 (4)	0.003 (3)	−0.002 (3)	−0.004 (3)
C24	0.072 (4)	0.087 (4)	0.064 (4)	0.005 (3)	0.004 (3)	−0.002 (3)
C25	0.071 (4)	0.096 (4)	0.079 (4)	0.000 (3)	0.010 (3)	0.010 (4)
C26	0.107 (5)	0.054 (3)	0.073 (4)	0.002 (3)	0.013 (3)	0.003 (3)
C27	0.086 (4)	0.090 (4)	0.058 (4)	−0.004 (3)	0.003 (3)	−0.009 (3)
C28	0.079 (5)	0.082 (4)	0.139 (6)	−0.014 (3)	−0.005 (4)	0.010 (4)
C29	0.110 (5)	0.067 (4)	0.081 (4)	0.000 (3)	−0.028 (4)	0.001 (3)
C30	0.090 (5)	0.087 (5)	0.138 (7)	−0.015 (4)	0.018 (5)	−0.018 (5)
C31	0.101 (5)	0.081 (4)	0.070 (4)	−0.014 (3)	−0.005 (3)	0.010 (3)
C32	0.148 (7)	0.106 (6)	0.076 (5)	−0.042 (5)	0.025 (5)	−0.005 (4)
C33	0.096 (5)	0.086 (4)	0.103 (5)	−0.021 (4)	0.049 (4)	−0.005 (4)
C34	0.103 (5)	0.093 (5)	0.111 (6)	−0.004 (4)	−0.020 (4)	0.035 (5)
C35	0.077 (5)	0.112 (6)	0.166 (8)	0.019 (4)	0.003 (5)	0.039 (6)
C36	0.085 (5)	0.139 (8)	0.202 (11)	0.034 (5)	0.022 (6)	0.079 (8)

### Geometric parameters (Å, °)

**Table:**

O1—C23	1.219 (7)	C12—C14	1.381 (8)
O2—C2	1.225 (6)	C12—C16	1.401 (9)
O3—C20	1.215 (6)	C13—H13	0.9300
O11—C6	1.376 (6)	C14—C18	1.399 (8)
O11—C24	1.427 (6)	C14—H14	0.9300
O4—C8	1.366 (6)	C16—C17	1.370 (9)
O4—C27	1.436 (7)	C16—H16	0.9300
O7—C18	1.372 (7)	C17—C26	1.518 (8)
O7—C22	1.427 (8)	C19—H19	0.9300
O8—C12	1.362 (7)	C21—C27	1.485 (9)
O8—C31	1.421 (8)	C21—H21A	0.9700
O5—C21	1.408 (7)	C21—H21B	0.9700
O5—C33	1.412 (7)	C22—C30	1.443 (9)
O6—C32	1.342 (8)	C22—H22A	0.9700
O6—C30	1.436 (8)	C22—H22B	0.9700
O9—C36	1.275 (9)	C23—C28	1.465 (9)
O9—C34	1.418 (8)	C23—C29	1.483 (8)
O10—C35	1.337 (9)	C24—C25	1.464 (8)
O10—C25	1.403 (8)	C24—H24A	0.9700
N2—C20	1.328 (6)	C24—H24B	0.9700
N2—C11	1.441 (6)	C25—H25A	0.9700
N2—H2	0.8600	C25—H25B	0.9700
N1—C2	1.316 (7)	C26—H26A	0.9700
N1—C26	1.452 (7)	C26—H26B	0.9700
N1—H1	0.8600	C27—H27A	0.9700
N3—C13	1.320 (7)	C27—H27B	0.9700
N3—C19	1.333 (7)	C28—H28A	0.9600
C1—C9	1.370 (7)	C28—H28B	0.9600
C1—C13	1.400 (7)	C28—H28C	0.9600
C1—C2	1.487 (7)	C29—H29A	0.9600
C3—C6	1.370 (7)	C29—H29B	0.9600
C3—C15	1.393 (7)	C29—H29C	0.9600
C3—H3	0.9300	C30—H30A	0.9700
C4—C9	1.381 (7)	C30—H30B	0.9700
C4—C19	1.384 (7)	C31—C34	1.500 (9)
C4—C20	1.498 (7)	C31—H31A	0.9700
C5—C15	1.369 (7)	C31—H31B	0.9700
C5—C8	1.381 (7)	C32—C33	1.477 (10)
C5—H5	0.9300	C32—H32A	0.9700
C6—C7	1.394 (7)	C32—H32B	0.9700
C7—C8	1.377 (7)	C33—H33A	0.9700
C7—H7	0.9300	C33—H33B	0.9700
C9—H9	0.9300	C34—H34A	0.9700
C10—C17	1.364 (8)	C34—H34B	0.9700
C10—C18	1.373 (8)	C35—C36	1.390 (11)
C10—H10	0.9300	C35—H35A	0.9700
C11—C15	1.503 (7)	C35—H35B	0.9700
C11—H11A	0.9700	C36—H36A	0.9700
C11—H11B	0.9700	C36—H36B	0.9700
			
C6—O11—C24	117.0 (4)	C30—C22—H22A	109.5
C8—O4—C27	118.0 (4)	O7—C22—H22B	109.5
C18—O7—C22	117.5 (5)	C30—C22—H22B	109.5
C12—O8—C31	119.2 (5)	H22A—C22—H22B	108.1
C21—O5—C33	111.8 (4)	O1—C23—C28	120.7 (6)
C32—O6—C30	112.6 (6)	O1—C23—C29	119.7 (6)
C36—O9—C34	122.1 (6)	C28—C23—C29	119.5 (7)
C35—O10—C25	119.5 (6)	O11—C24—C25	109.7 (5)
C20—N2—C11	121.2 (4)	O11—C24—H24A	109.7
C20—N2—H2	119.4	C25—C24—H24A	109.7
C11—N2—H2	119.4	O11—C24—H24B	109.7
C2—N1—C26	119.6 (5)	C25—C24—H24B	109.7
C2—N1—H1	120.2	H24A—C24—H24B	108.2
C26—N1—H1	120.2	O10—C25—C24	107.4 (5)
C13—N3—C19	115.9 (4)	O10—C25—H25A	110.2
C9—C1—C13	116.6 (5)	C24—C25—H25A	110.2
C9—C1—C2	125.4 (4)	O10—C25—H25B	110.2
C13—C1—C2	118.0 (5)	C24—C25—H25B	110.2
O2—C2—N1	121.9 (5)	H25A—C25—H25B	108.5
O2—C2—C1	120.3 (5)	N1—C26—C17	111.8 (5)
N1—C2—C1	117.7 (4)	N1—C26—H26A	109.3
C6—C3—C15	118.9 (5)	C17—C26—H26A	109.3
C6—C3—H3	120.5	N1—C26—H26B	109.3
C15—C3—H3	120.5	C17—C26—H26B	109.3
C9—C4—C19	117.5 (5)	H26A—C26—H26B	107.9
C9—C4—C20	124.1 (4)	O4—C27—C21	108.2 (5)
C19—C4—C20	118.3 (5)	O4—C27—H27A	110.1
C15—C5—C8	120.5 (5)	C21—C27—H27A	110.1
C15—C5—H5	119.8	O4—C27—H27B	110.1
C8—C5—H5	119.8	C21—C27—H27B	110.1
C3—C6—O11	124.3 (5)	H27A—C27—H27B	108.4
C3—C6—C7	121.3 (5)	C23—C28—H28A	109.5
O11—C6—C7	114.3 (4)	C23—C28—H28B	109.5
C8—C7—C6	118.8 (5)	H28A—C28—H28B	109.5
C8—C7—H7	120.6	C23—C28—H28C	109.5
C6—C7—H7	120.6	H28A—C28—H28C	109.5
O4—C8—C7	124.6 (5)	H28B—C28—H28C	109.5
O4—C8—C5	115.1 (5)	C23—C29—H29A	109.5
C7—C8—C5	120.2 (5)	C23—C29—H29B	109.5
C1—C9—C4	120.3 (5)	H29A—C29—H29B	109.5
C1—C9—H9	119.9	C23—C29—H29C	109.5
C4—C9—H9	119.9	H29A—C29—H29C	109.5
C17—C10—C18	119.6 (6)	H29B—C29—H29C	109.5
C17—C10—H10	120.2	O6—C30—C22	110.1 (6)
C18—C10—H10	120.2	O6—C30—H30A	109.6
N2—C11—C15	110.6 (4)	C22—C30—H30A	109.6
N2—C11—H11A	109.5	O6—C30—H30B	109.6
C15—C11—H11A	109.5	C22—C30—H30B	109.6
N2—C11—H11B	109.5	H30A—C30—H30B	108.2
C15—C11—H11B	109.5	O8—C31—C34	108.5 (6)
H11A—C11—H11B	108.1	O8—C31—H31A	110.0
O8—C12—C14	124.9 (6)	C34—C31—H31A	110.0
O8—C12—C16	115.3 (6)	O8—C31—H31B	110.0
C14—C12—C16	119.7 (6)	C34—C31—H31B	110.0
N3—C13—C1	125.3 (5)	H31A—C31—H31B	108.4
N3—C13—H13	117.4	O6—C32—C33	114.0 (6)
C1—C13—H13	117.4	O6—C32—H32A	108.8
C12—C14—C18	118.4 (5)	C33—C32—H32A	108.8
C12—C14—H14	120.8	O6—C32—H32B	108.8
C18—C14—H14	120.8	C33—C32—H32B	108.8
C5—C15—C3	120.2 (5)	H32A—C32—H32B	107.7
C5—C15—C11	120.5 (5)	O5—C33—C32	111.5 (6)
C3—C15—C11	119.2 (5)	O5—C33—H33A	109.3
C17—C16—C12	120.2 (6)	C32—C33—H33A	109.3
C17—C16—H16	119.9	O5—C33—H33B	109.3
C12—C16—H16	119.9	C32—C33—H33B	109.3
C10—C17—C16	120.7 (6)	H33A—C33—H33B	108.0
C10—C17—C26	119.7 (6)	O9—C34—C31	110.5 (6)
C16—C17—C26	119.7 (6)	O9—C34—H34A	109.6
O7—C18—C10	124.1 (6)	C31—C34—H34A	109.6
O7—C18—C14	114.5 (5)	O9—C34—H34B	109.6
C10—C18—C14	121.3 (5)	C31—C34—H34B	109.6
N3—C19—C4	124.4 (5)	H34A—C34—H34B	108.1
N3—C19—H19	117.8	O10—C35—C36	119.9 (7)
C4—C19—H19	117.8	O10—C35—H35A	107.4
O3—C20—N2	122.4 (5)	C36—C35—H35A	107.4
O3—C20—C4	121.0 (4)	O10—C35—H35B	107.4
N2—C20—C4	116.5 (4)	C36—C35—H35B	107.4
O5—C21—C27	109.0 (5)	H35A—C35—H35B	106.9
O5—C21—H21A	109.9	O9—C36—C35	122.4 (7)
C27—C21—H21A	109.9	O9—C36—H36A	106.7
O5—C21—H21B	109.9	C35—C36—H36A	106.7
C27—C21—H21B	109.9	O9—C36—H36B	106.7
H21A—C21—H21B	108.3	C35—C36—H36B	106.7
O7—C22—C30	110.6 (6)	H36A—C36—H36B	106.6
O7—C22—H22A	109.5		
			
C26—N1—C2—O2	4.6 (9)	C18—C10—C17—C26	−177.6 (5)
C26—N1—C2—C1	−174.1 (5)	C12—C16—C17—C10	−1.2 (8)
C9—C1—C2—O2	164.7 (5)	C12—C16—C17—C26	177.4 (5)
C13—C1—C2—O2	−15.0 (7)	C22—O7—C18—C10	−4.1 (8)
C9—C1—C2—N1	−16.6 (7)	C22—O7—C18—C14	173.9 (5)
C13—C1—C2—N1	163.6 (5)	C17—C10—C18—O7	177.4 (5)
C15—C3—C6—O11	177.6 (4)	C17—C10—C18—C14	−0.4 (8)
C15—C3—C6—C7	−0.3 (7)	C12—C14—C18—O7	−178.0 (5)
C24—O11—C6—C3	−14.7 (7)	C12—C14—C18—C10	0.0 (8)
C24—O11—C6—C7	163.3 (4)	C13—N3—C19—C4	1.7 (7)
C3—C6—C7—C8	2.6 (7)	C9—C4—C19—N3	−1.2 (7)
O11—C6—C7—C8	−175.4 (4)	C20—C4—C19—N3	−179.7 (4)
C27—O4—C8—C7	−11.6 (7)	C11—N2—C20—O3	−2.1 (8)
C27—O4—C8—C5	166.1 (5)	C11—N2—C20—C4	−179.0 (4)
C6—C7—C8—O4	174.7 (4)	C9—C4—C20—O3	−157.1 (5)
C6—C7—C8—C5	−2.9 (7)	C19—C4—C20—O3	21.3 (7)
C15—C5—C8—O4	−176.9 (4)	C9—C4—C20—N2	19.9 (7)
C15—C5—C8—C7	0.9 (7)	C19—C4—C20—N2	−161.7 (4)
C13—C1—C9—C4	0.3 (7)	C33—O5—C21—C27	−178.5 (6)
C2—C1—C9—C4	−179.4 (4)	C18—O7—C22—C30	−165.1 (5)
C19—C4—C9—C1	0.1 (7)	C6—O11—C24—C25	−159.8 (5)
C20—C4—C9—C1	178.5 (4)	C35—O10—C25—C24	167.0 (8)
C20—N2—C11—C15	175.5 (5)	O11—C24—C25—O10	69.1 (7)
C31—O8—C12—C14	−10.5 (8)	C2—N1—C26—C17	−163.6 (5)
C31—O8—C12—C16	166.9 (5)	C10—C17—C26—N1	80.1 (7)
C19—N3—C13—C1	−1.3 (8)	C16—C17—C26—N1	−98.6 (6)
C9—C1—C13—N3	0.3 (7)	C8—O4—C27—C21	−162.2 (5)
C2—C1—C13—N3	−179.9 (5)	O5—C21—C27—O4	75.9 (6)
O8—C12—C14—C18	177.1 (5)	C32—O6—C30—C22	−123.8 (8)
C16—C12—C14—C18	−0.2 (8)	O7—C22—C30—O6	73.0 (8)
C8—C5—C15—C3	1.4 (7)	C12—O8—C31—C34	−162.0 (5)
C8—C5—C15—C11	177.2 (4)	C30—O6—C32—C33	−177.1 (6)
C6—C3—C15—C5	−1.8 (7)	C21—O5—C33—C32	−160.8 (6)
C6—C3—C15—C11	−177.5 (4)	O6—C32—C33—O5	−66.5 (10)
N2—C11—C15—C5	−97.7 (6)	C36—O9—C34—C31	−133.9 (10)
N2—C11—C15—C3	78.0 (6)	O8—C31—C34—O9	71.5 (8)
O8—C12—C16—C17	−176.7 (5)	C25—O10—C35—C36	177.2 (9)
C14—C12—C16—C17	0.8 (8)	C34—O9—C36—C35	−168.2 (10)
C18—C10—C17—C16	1.0 (8)	O10—C35—C36—O9	−34.3 (17)

### Hydrogen-bond geometry (Å, °)

**Table:**

*D*—H···*A*	*D*—H	H···*A*	*D*···*A*	*D*—H···*A*
N1—H1···O1	0.86	2.36	3.209 (7)	170.
N2—H2···O1	0.86	2.53	3.382 (7)	172.
C9—H9···O1	0.93	2.43	3.271 (7)	151.
C3—H3···O11^i^	0.93	2.46	3.356 (6)	161.
C33—H33B···N3^ii^	0.97	2.60	3.401 (8)	140.

Symmetry codes: (i) −*y*+2, *x*, *z*−1/4; (ii) −*y*+1, *x*, *z*+3/4.
